# Approximate matrix exponential: a fast and accurate solver for Markov kinetic models of ion channels

**DOI:** 10.3389/fncom.2026.1901593

**Published:** 2026-10-06

**Authors:** David Alton McDougall

**Affiliations:** Independent Researcher, Nashua, NH, United States

**Keywords:** ion channel, kinetic model, Markov model, NEURON, neuronal simulation, numerical integration

## Abstract

A new method is presented for simulating Markov kinetic models of ion channels, which are biologically realistic but difficult to simulate. Computing the analytic solution is prohibitively expensive, necessitating the use of numerical methods. The new method creates a piecewise polynomial approximation of the propagator matrix, which is the analytic solution operator. The new method is an order of magnitude faster and more accurate than previous numerical methods, but it is limited to models with one or two input variables and its time complexity scales quadratically with the number of model states. The accompanying program, *ame*, applies the new method to the NEURON simulator. For comparison, the analytic solution was also added to NEURON. The new method achieves real-time performance on a state-of-the-art GPU for tens to hundreds of thousands of model instances. This method will allow large-scale neuronal simulations to use more detailed models of ion channels.

## 1 Introduction

Neurons are composed of many components and so realistic computer models of neurons are likewise composed of many software components. Scientists making computer models of the brain face trade-offs between the quantity and the quality of their components. Simplified models of neurons are not faithful to biology but are easy to simulate in large numbers. In contrast, conductance-based models of neurons are biologically realistic but are painfully time-consuming to simulate. This article introduces a new tool for simulating Markov kinetic models of ion channels, which are the most numerous component of conductance-based models.

Markov models of molecular kinetics are at the foundation of neuroscience, where they describe the physical components that implement the brain's computations (Destexhe et al., 1994). They were first applied to neuroscience by Hodgkin and Huxley (1952) to describe the ion channels responsible for generating action potentials. Because brains are composed of trillions of repeated components, computational neuroscience has a voracious appetite for Markov kinetic models.

Ion channels are the macromolecules responsible for conducting electrically charged ions across neuronal membranes. The conductance of an ion channel is controlled by its conformational state. The molecular dynamics of an isolated ion channels can be modeled as a piecewise-deterministic Markov process (Davis, 1984). To enable longer time-scale simulations, the space of conformational states is discretized, which yields a Markovian jump process between a finite number of discrete conformational states (Sarich et al., 2014; Schwantes et al., 2014). For neuronal simulations, time is also discretized, yielding a discrete-time Markov chain.

The molecules of interest to neuroscience have a large number of physically distinct states. However, many of those states are functionally indistinguishable from each other (Balbi et al., 2017; Catacuzzeno et al., 2024). In contrast, computational models, such as Hodgkin-Huxley style models, have very few states, which allows for fast simulation, but consequently they lack important features such as desensitization (Robert and Howe, 2003; Andreozzi et al., 2019). Most phenomena of interest to neuroscience can be modeled with a dozen states or less.

Markov kinetic models of ion channels are created in the following manner. First, ion channels are isolated and their electric and chemical properties are measured (Ranjan et al., 2019). Next, the kinetic scheme is designed, i.e., the number of states, their connectivity, and the form of the state transition rate functions (Destexhe et al., 1998; Menon et al., 2009). Finally, the parameters of the model are optimized until its outputs match the experimentally collected data. Although this process does not model molecular dynamics, it can accurately reproduce the macroscopic conductance of ion channels.

By modeling probabilities, deterministic equations can capture the stochastic nature of the underlying physical process. The state of a Markov chain, *s*, represents a probability distribution over the model's states. Markov chains are defined by the master Equation 1, which describes how the state evolves over time. *Q* is the transition rate matrix. The initial value problem of finding *s*(*t*) given *s*(0) = *s*_0_ has an analytic solution (Equation 2). Reformulating the solution yields *P*, the transition probability matrix (Equation 3), which is also known as the *propagator* matrix. The propagator matrix advances the state of the model over time (Equation 4).


$$\begin{array}{l} \frac{ds}{dt}=Qs \end{array}$$ (1)

$$\begin{array}{l} s\left(t\right)=e^{Qt}s_{0} \end{array}$$ (2)

$$\begin{array}{l} P\left(t\right)=e^{Qt} \end{array}$$ (3)

$$\begin{array}{l} s\left(t\right)=P\left(t\right)s_{0} \end{array}$$ (4)

Neuronal simulations repeatedly advance models over a fixed time step, so the integration period *t* may be replaced by the time step of the simulation, Δ*t*, which is constant. Models receive an input vector, *i*, which is a function of time and controls the model dynamics. For neuronal simulations, the input vector is assumed to be constant over the duration of each time step, and to only change at time step boundaries. The master equation is then Equation 5 and it follows that the propagator matrix function is Equation 6 and the model advances via Equation 7.


$$\begin{array}{l} \frac{ds}{dt}=Q\left(i\right)s \end{array}$$ (5)

$$\begin{array}{l} P\left(i\right)=e^{Q\left(i\right)\Delta t} \end{array}$$ (6)

$$\begin{array}{l} s_{\Delta t}\left(i\right)=P\left(i\right)s_{0} \end{array}$$ (7)

For some models, an explicit symbolic representation of *P* can be found (Rotter and Diesmann, 1999). In general, *P* is expressed in terms of the matrix exponential function. There are many methods of calculating the matrix exponential (Moler and Van Loan, 1978). Direct evaluation of its Taylor series expansion converges slowly and is numerically unstable. Alternative methods have been developed, such as scaling and squaring with Padé approximants (Higham, 2005), which performs well with small dense matrices, and the state of the art continues to improve in both speed and accuracy (Al-Mohy and Higham, 2009; Ruiz et al., 2016; Blanes et al., 2025; Sastre et al., 2026). For neuronal simulations, *P* and *Q* are small dense matrices. Calculating the matrix exponential inevitably involves matrix-matrix multiplication, whose time complexity is cubic with respect to the order of the matrix.

Established numerical methods find approximate solutions to the initial value problem Equation 5 using only *Q*, the transition rate matrix. Numerical methods fall into two categories: explicit and implicit:

- Explicit integration methods work by evaluating the derivative (*Q*) and running the state forward in time. They are simple to apply and efficient to compute. Their time-complexity is quadratic with respect to the number of model states. They can be numerically unstable, which is when small errors accumulate and compound exponentially, leading to sudden catastrophic failure. Examples of explicit methods include the forward Euler and Runge–Kutta–Chebyshev methods (Kobayashi et al., 2023).
- Implicit integration methods work by evaluating the derivative at the end of the time step (*Q*_*t* + Δ*t*_). Implicit methods offer accurate and numerically stable integration. They have cubic time complexity with respect to the number of model states because they require solving a system of equations via Gaussian elimination. Examples of implicit methods include the backward Euler and Crank–Nicolson methods. They are considered state of the art and the NEURON simulator uses them by default (Carnevale and Hines, 2006).
  – Technically, the Crank–Nicolson method is semi-implicit. It advances by averaging the derivative at the start and end of the time step (Crank and Nicolson, 1947). It has cubic time complexity for the same reason implicit methods do. It is unconditionally stable and its error is bounded by *O*(Δ*t*^2^). For small values of Δ*t* it is significantly more accurate than the backward Euler method. However, it exhibits damped oscillations and for large values of Δ*t* it is less accurate than the backward Euler method, whose error is bounded by *O*(Δ*t*) (Mascagni and Sherman, 1998).

## 2 Methods

The approximate matrix exponential (AME) method integrates Markov models by approximating the propagator matrix function (Equation 6) to advance the model state (Equation 7), for example see Figure 1. This section describes how the AME method makes and uses the approximation.

**Figure 1:**
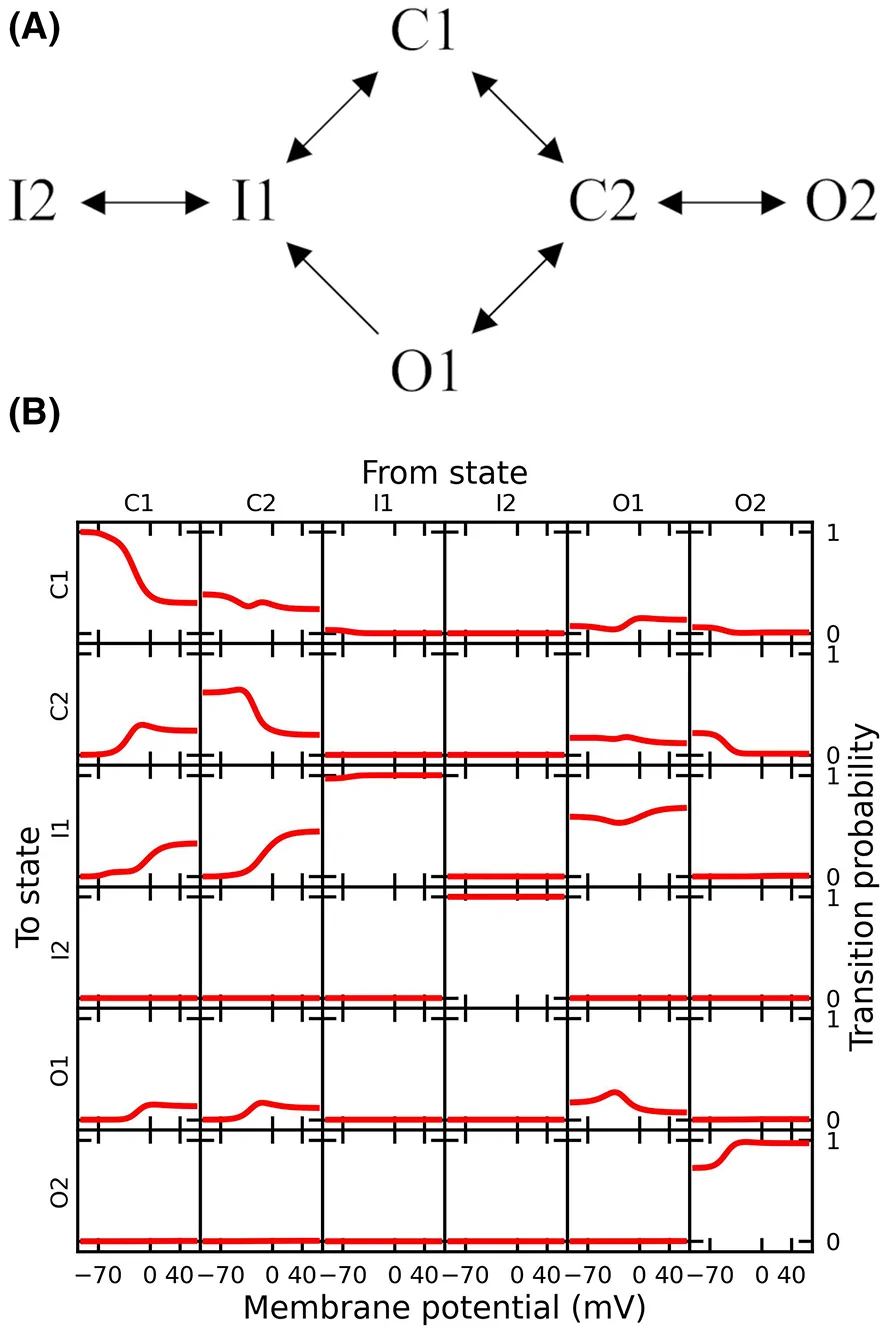
Example model of *Na*_*v*_1.1 voltage gated sodium ion channel (Balbi et al., 2017). **(A)** Kinetic scheme. The model has six states: closed (C1 & C2), inactive (I1 & I2), and open (O1 & O2). The channel conducts sodium ions in the open states. The state-transition rates are functions of the membrane's electric potential (equations omitted). Adapted from (Balbi et al., 2017), licensed under CC BY 4.0. **(B)** Propagator matrix. This 6 × 6 matrix describes the probability of transitioning from every state to every other state, as a function of membrane potential, after 0.025 ms, and at 37 °C. This article explains how to build a fast and accurate approximation of the propagator matrix function.

### 2.1 Building the approximation

The AME method constructs a piecewise polynomial approximation of the propagator matrix function. The approximation is structured as follows: the input space is partitioned into a regular grid of non-overlapping regions, each containing a polynomial approximation of the function in that region. Within each region, the input vector is transformed into the range 0 ≤ *i* ≤ 1. The polynomials are fit to sampled data using a linear least squares regression.

The minimum number of samples required in each region for each the regression is the number of free parameters in the polynomial with a safety factor of 100×. Samples for each region are drawn from a stratified distribution, which partitions the sample space into a regular grid of strata and draws at least one sample from each strata. A database of samples is collected, updated, and sampled from as needed.

Since the function being approximated returns a matrix, the AME method constructs an approximation for each cell of the matrix. For computational efficiency, all approximations for a matrix share the same input space partitions and polynomial form.

### 2.2 Measuring the accuracy

The approximation's accuracy is quantified by its integration error, which is defined as the maximum absolute error in any component of the state vector after integrating over one unit of time, over all possible initial states and inputs. The properties of Markov models allow this to be measured efficiently (Stewart, 1994).

As a result of the Chapman–Kolmogorov equation, the product of two propagator matrices is also a propagator matrix whose integration period is the sum of its factors' integration periods. For example, squaring a propagator matrix doubles its integration period. The AME method uses this property to increase the integration period of the samples and the approximation from one time step to one unit of time.

Markov models are linear. Therefore, the error in each component of the state vector is a weighted linear combination of the errors in that component's corresponding row of the approximated propagator matrix. The weights of the linear combination are the components of state vectors, which represent probability distributions. Because probability is a conserved quantity (i.e., state vectors are non-negative and sum to one), we can infer that the maximum error will occur when 100% of the model is in a single state. It follows that the error of the standard basis vectors is the maximum error of all possible state vectors.

As a result, the integration error for an input sample is Equation 8, where *p* is the approximated propagator matrix, and *P* is the exact propagator matrix.


$$\begin{array}{l} \text{max}\left(\left|p{\left(i\right)}^{\text{round}\left(1/\text{Δ}\text{t}\right)}-P{\left(i\right)}^{\text{round}\left(1/\text{Δ}\text{t}\right)}\right|\right) \end{array}$$ (8)

A true and unbiased estimate of the accuracy would require generating new samples at great expense. Instead, the samples that were used to construct the approximation itself are reused to calculate the *residual* integration error.

### 2.3 Controlling the accuracy

The AME method controls the accuracy of the approximation. The integration error is monotonically related to the size of the input partitions. Therefore, it is trivial and efficient for a greedy algorithm to find input space partitions that yield a user-specified level of accuracy, known as the *error target*.

With one input dimension, the procedure for determining the number of partitions satisfies the error target with the fewest partitions possible. The procedure works by designating an input space partitioning as the *cursor* and walking the cursor through the space of all possible partitionings to find an optimal value. With one input dimension, the cursor starts at 20 and increases by 50% on each step until it's residual integration error crosses below the error target, at which point it switches to a bisecting search to find the smallest cursor whose residual integration error is less than or equal to the error target.

With two input dimensions, a different greedy algorithm is employed, which satisfies the error target but does not globally minimize the number of partitions. With two input dimensions, the cursor starts at (4, 4). The cursor tries increasing each dimension by 50% and moves along the dimension that yields the minimum residual integration error. Fractional increases are rounded up. This repeats until advancing the cursor would cause its error to cross below the error target, at which point the rate of increase is halved and it continues from the previous cursor. This repeats until the increases are reduced to the minimum possible increase of 1, at which point the cursor advances by 1 and is returned as the solution.

If the relationship between the number of partitions and the resulting integration error is found to be non-monotonic then the propagator matrix function is numerically unstable and the model must be either rejected or retried with a larger error target.

However, when applied to excessively large intervals, high-degree polynomial approximations yield unpredictable results, resulting in a non-monotonic error landscape. Enforcing the constraint that the propagator matrix is stochastic restores monotonicity. When calculating the residual integration error for two-input models, the AME method clips the approximated propagator matrix to the range [0, 1] and divides each column by its sum.

### 2.4 Optimizing for speed

Now let's discuss how to build the fastest approximation. The approximation has two types of free parameter: the input space partitions and the form of the polynomial, both of which impact both the speed and accuracy of the approximation. The input space partitions are already controlling the accuracy, leaving us with only one type of free parameter and only one target for control: we will optimize the form of the polynomial for speed.

The speed of the approximation is quantified as the marginal time to advance per instance per time step, i.e., the throughput. The AME method measures the speed by integrating large batches of randomly initialized state and input vectors. To reduce measurement variability, each measurement is repeated 20 times and the minimum elapsed time is reported. To eliminate fixed overhead, the difference in the time to advance a batch of 10,000 instances vs. a batch of 110,000 instances is measured. When running on graphical processing units (GPUs), batches of 100,000 and 1,100,000 instances are advanced instead.

This paragraph explains how the AME method finds the best polynomial. It uses a hill climbing algorithm to explores the space of polynomial forms and find the first local maxima of performance. The algorithm is performed as follows. First an initial polynomial form is designated as the *cursor*. The cursor polynomial is used to make an approximation that meets the error target, as described in Section 2.3. Then it measures the speed of the cursor and the speed of all polynomial forms that are adjacent to the cursor. Polynomials are adjacent if they differ by one term. If the cursor is faster than all adjacent forms then the cursor is returned as the solution. If the cursor is not the fastest, then the fastest polynomial form is re-designate as the new cursor and the procedure is repeated.

The initial univariate polynomial form is *x*^3^ + *x*^2^ + *x* + 1. The initial bivariate polynomial form is *x*^3^ + *y*^3^ + *x*^2^ + *y*^2^ + *xy* + *x* + *y* + 1. The space of allowable polynomials is constrained: univariate polynomials must be complete, and bivariate polynomials must satisfy the property that for each term *x*^*A*^*y*^*B*^ there must also exist terms *x*^*a*^*y*^*b*^ for each *a* ∈ [0, *A*], *b* ∈ [0, *B*].

### 2.5 Chemical concentrations

The effects of chemical concentrations span many orders of magnitude, which is difficult to approximate using fixed-size piecewise regions because the necessary region size may be orders of magnitude smaller than the data range. The AME method preconditions inputs that represent chemical concentrations using Equation 9, where ϵ is a small positive offset which shifts the domain of the transform to include zero.


$$\begin{array}{l} i^{\prime }=\log_{2}\left(i+\epsilon \right) \end{array}$$ (9)

The AME method determines the best offset ϵ experimentally. It tries 50 offsets in a geometric progression spanning from 10^−9^ mM to *i*_*max*_. It builds an approximation for each offset and measures the root-mean-square of the residual errors for each approximation. The optimal offset ϵ minimizes this measurement. If either 10^−9^ mM or *i*_*max*_ are the global minimum, then the best offset may be outside the span of offsets searched by this procedure, and so an error is raised to the user. All approximations use the same input partitions and polynomial form: 20 and *x*^3^ + *x*^2^ + *x* + 1, respectively for one input dimension; (20, 20) and *x*^3^ + *y*^3^ + *x*^2^ + *y*^2^ + *xy* + *x* + *y* + 1, respectively for two input dimensions. The optimal offset is independent of those parameters. For consistency, all required samples of the propagator matrix are gathered before beginning this procedure. This procedure is performed before determining the optimal polynomial form.

The base 2 logarithm is preferred because it can be slightly faster to compute than other bases of logarithm. Efficient algorithms exist for calculating log_2_ using binary number representation. Some computer systems have dedicated instructions for calculating log_2_, for example the lg2 instruction for Nvidia PTX assembly. Other bases can be derived from log_2_ via a change of base: log_*x*_(*y*) = log_2_(*y*)/log_2_(*x*). Therefore, log_2_ is at most one floating-point multiplication faster to compute.

### 2.6 Preserving the stochastic structure

The state vector represents a probability distribution, which implies that its components are non-negative and sum to one. The AME method preserves the sum of the state components by dividing the state vector by its sum. This correction is applied immediately after integration. Without this correction, the sum would drift in random directions at a rate controlled by the error target.

The AME method does not enforce the constraint that state components are in the range [0, 1]. States can go negative, but they cancel out when they interact with positive states, so negative states should not persist indefinitely.

### 2.7 Performance considerations

The following implementation details are important for fast running simulations:

- All model instances must be integrated in a single batch, which allows the computer's data cache to accelerate repeated memory accesses. In particular, reading the polynomial coefficients is a major performance bottleneck. The coefficients are loaded into the computer's cache once per batch, so larger batches are more efficient.
- The polynomial coefficients within each region must be arrange in memory such that they are accessed sequentially and contiguously because caches are optimized for accessing adjacent memory locations.
- Each cell of the propagator matrix must be used as soon as it is computed. This means that the matrix-vector multiplication of the propagator matrix and the state vector must be incrementally computed as each cell of the propagator matrix becomes available.
- The procedure for integration (Section 2.8), which is the run-time portion of the AME method, must be implemented using a compiled programming language, such as C/C++.
- Template-based source code generation must be used to insert the following values into for the procedure for integration's source code before compilation. This allows the compiler to optimize the program more aggressively. The source code begins as a template, which data are inserted into to generate a complete source-code file, which is then compiled and executed. The following information is inserted into the template:
  – *N*, which is the number of model states.
  – *D*, which is the number of input dimensions.
  – *i*_*min*_ and *i*_*max*_, which are vectors in ℝ^*D*^ that define the input domain as *i*_*min*_ ≤ *i* ≤ *i*_*max*_.
  – *IsChemical*, which is a vector in 𝔹^*D*^ that indicates which input dimensions represent chemical concentrations.
  – ϵ, which is a vector in ${\mathbb{R}}_{>0}^{D}$ containing the logarithmic offset in each input dimension. Dimensions which do not represent chemical concentrations have an undefined value and are never read.
  – *R*, which is a vector in ℕ^*D*^ containing the number of piecewise regions in each input dimension.
  – *T*, which is the number of terms in each polynomial approximation.
  – *basis*(*i*), which is a function ℝ^*D*^ → ℝ^*T*^ that accepts the polynomial's independent variables and computes the unscaled terms of the polynomial (the basis vector). Polynomials are evaluated as the dot product of a basis vector and a coefficient vector.
  – *C*, which is the array of all polynomial coefficients, for every region and every cell of the propagator matrix. This array has the shape (*R*_1_, ..., *R*_*D*_, *N, N, T*) and is indexed as *C*_*region,column,row,term*_ with zero-based indices. It is stored in *row-major* format so that the vector of coefficients for each polynomial is stored contiguously in the final dimension of the array.

### 2.8 Procedure for integration

1. The input and state vectors are read from memory into local variables *i* and *s*, respectively.
2. Inputs that fall outside of the input domain are clipped into the range *i*_*min*_ ≤ *i* ≤ *i*_*max*_. This step handles out-of-bounds inputs inaccurately. Alternatively, the procedure could abort and return an error message.

 *i* ← max(*i*_*min*_, min(*i*_*max*_, *i*))
3. Inputs that represent chemical concentrations are transformed, as explained in Section 2.5. Likewise, *i*_*min*_ and *i*_*max*_ are transformed so that *i* remains in the range *i*_*min*_ ≤ *i* ≤ *i*_*max*_.

 **for** *x* **in** [1, *D*] **do**
   **if** *IsChemical*_*x*_ **then**
      *i*_*x*_ ← log_2_(*i*_*x*_ + ϵ_*x*_)
      *i*_*min*_*x*__ ← log_2_(*i*_*min*_*x*__ + ϵ_*x*_)
      *i*_*max*_*x*__ ← log_2_(*i*_*max*_*x*__ + ϵ_*x*_)
   **end if**
 **end for**
4. The input vector is transformed into *r*, a coordinate vector in the region space: 0 ≤ *r* ≤ *R*. Then *r* is split into whole and fractional parts, which represent the index of the region containing the input vector, and the input vector's location within the region, respectively.

 *r* ← (*i* − *i*_*min*_)/(*i*_*max*_ − *i*_*min*_)*R*
 *r*_*whole*_ ← ⌊*r*⌋
 *r*_*fraction*_ ← *r* − *r*_*whole*_
5. Regions cover half-open intervals except for the final region along each dimension, which is closed at its upper boundary. A special case extends the final regions to include the maximum input values.

 **for** *x* **in** [1, *D*] **do**
   **if** *r*_*whole*_*x*__ = *R*_*x*_ **then**
      *r*_*whole*_*x*__ ← *R*_*x*_ − 1
      *r*_*fraction*_*x*__ ← 1
   **end if**
 **end for**
6. The input vector's location within the piecewise region is used to calculate *b*, the basis vector, which contains the unscaled terms of the polynomial.

  *b* ← *basis*(*r*_*fraction*_)
7. A new state vector is allocate and zeroed, which will accumulate the incremental results of the matrix-vector multiplication.

  *s*′ ← 0
8. Each polynomial approximation is evaluated as the dot product of its coefficient vector and the basis vector, resulting in a single cell of the propagator matrix, which is immediately multiplied with a component of the original state vector and added to the new state vector. This operation incrementally computes the product of the propagator matrix and the state vector.

 **for** *column* **in** [1, *N*] **do**
       **for** *row* **in** [1, *N*] **do**
     $s_{row}^{\prime }\leftarrow s_{row}^{\prime }+\left(b\cdot C_{r_{whole},column-1,row-1,:}\right)\;s_{column}$

      **end for**
 **end for**
9. The new state vector is normalized by dividing it by its sum so that the total probability is conserved, as explained in Section 2.6.
   $s^{\prime }\leftarrow \frac{s^{\prime }}{\Sigma_{x=1}^{N}s_{x}^{\prime }}$
10. The new state vector is written to memory.

  *s* ← *s*′

## 3 Results

### 3.1 Exact integration in NEURON

NEURON is a state-of-the-art simulator for conductance- based models of neurons. Previously it relied on the backward Euler method to simulate Markov kinetic models of ion channels.

Version 9.1 will introduce the *matexp* solver method to the NMODL language, which gives the analytic solution. This feature is implemented for the *nmodl-transpiler*, but not for *nocmodl*. It solves KINETIC blocks that are linear and time-invariant by computing the propagator matrix (Equation 6) at every time step and for every instance of the kinetic model.

Computing the matrix exponential dominates the speed and accuracy of this method. NEURON uses the Eigen library's implementation (Niesen and He, 2010) of Higham's (2005) algorithm, which is accurate to within unit round-off (Higham, 2005).

This method is useful because it is exact, and so it serves as an excellent reference for studying approximations. Exact solutions also offer peace of mind that approximations struggle to provide.

### 3.2 Fast integration in NEURON

The *ame* program implements the AME method for NEURON. This command-line program operates on NMODL files containing Markov kinetic models, which must be inside of KINETIC blocks. It outputs new NMODL files that are solved and ready for the *nrnivmodl* program to compile.

The ame program requires the time step and temperature. These values must be known ahead of time and they must not change thereafter. Otherwise they would be arguments to the propagator matrix function. Instead the time step and temperature are held constant and baked into the approximation.

The ame program also requires the input domain. The approximation is built to span the input domain. At simulation time, out-of-bounds inputs are clipped to the domain. Only one or two inputs are allowed.

This program uses the SciPy library's implementation of Al-Mohy and Higham's (2009) algorithm for calculating the matrix exponential function, which improves Higham's (2005) algorithm in both speed and accuracy (Al-Mohy and Higham, 2009).

The ame program is open source (under the MIT license) and available on PyPI, the Python Package Index. It was developed for Linux. To install the ame program, execute the command:

$ pip install matexp

### 3.3 Benchmarking the methods

The following sections measure the speed and accuracy of the backward Euler (bE), matrix exponential (ME), and approximate matrix exponential (AME) methods. Because these measurements depend on the specific model being simulated, they were performed on a benchmark of models (see Table 1).

**Table 1:** Markov models of ion channels for evaluating integration methods.

Channel	Inputs	States	References
AMPA	glu	13	Balmer et al., 2021
NMDA	v, glu	10	Kampa et al., 2004
Na_*v*_1.1	v	6	Balbi et al., 2017
Na_*v*_1.2	v	6	Balbi et al., 2017
Na_*v*_1.3	v	6	Balbi et al., 2017
Na_*v*_1.4	v	6	Balbi et al., 2017
Na_*v*_1.5	v	6	Balbi et al., 2017
Na_*v*_1.6	v	6	Balbi et al., 2017
Na_*v*_1.7	v	6	Balbi et al., 2017
Na_*v*_1.8	v	6	Balbi et al., 2017
Na_*v*_1.9	v	6	Balbi et al., 2017
K_*v*_1.1	v	4, 6, 11, 13	Johner, 2017
K_*v*_1.2	v	4, 6, 11, 13	Johner, 2017
K_*v*_1.3	v	4, 6, 11, 13	Johner, 2017
K_*v*_1.4	v	4, 6, 11, 13	Johner, 2017
K_*v*_1.5	v	4, 6, 11, 13	Johner, 2017
K_*v*_1.6	v	4, 6, 11, 13	Johner, 2017
K_*v*_1.7	v	4, 6, 11, 13	Johner, 2017
K_*v*_2.1	v	4, 11	Johner, 2017
K_*v*_2.2	v	4, 6, 11, 13	Johner, 2017
K_*v*_3.1	v	4, 6, 11, 13	Johner, 2017
K_*v*_3.2	v	4, 6, 11, 13	Johner, 2017
K_*v*_3.3	v	4, 6, 11, 13	Johner, 2017
K_*v*_3.4	v	4, 6, 11, 13	Johner, 2017
K_*v*_4.1	v	4, 6, 11, 13	Johner, 2017
K_*v*_4.2	v	4, 6, 11, 13	Johner, 2017
K_*v*_4.3	v	4, 6, 11, 13	Johner, 2017
K_*v*_6.4	v	4, 6, 11, 13	Johner, 2017
K_*v*_9.1	v	4, 6, 11, 13	Johner, 2017
K_*v*_10.1	v	4, 6, 11, 13	Johner, 2017
K_*v*_10.2	v	4, 6, 11, 13	Johner, 2017
K_*v*_12.1	v	4, 11	Johner, 2017
K_*v*_12.3	v	4, 6, 11, 13	Johner, 2017
KCNQ5	v	4, 6, 11, 13	Johner, 2017

This dataset contains seven kinetic schemes, some of which are reused for multiple subtypes of ion channel. These models were developed by four independent sources, each using different experimental data and parameter optimization software.

Four independent sources provided models for the benchmark. The first two sources, Balmer et al. (2021) and Kampa et al. (2004), modeled AMPA and NMDA receptors, respectively. Both recorded electrophysiological data, designed kinetic schemes, and fit parameters using the AxoGraph software. The third source, Balbi et al. (2017), designed a single kinetic scheme for all nine subtypes of voltage-gated Sodium channel (Na_*v*_). They collected electrophysiological data from the literature and fit parameters using the principal axis (PRAXIS) method which is built into NEURON. The fourth source, Johner (2017), applied 4 kinetic schemes to 23 subtypes of voltage-gated potassium channel (K_*v*_), yielding 88 models. Their data was provided by the Blue Brain Project, who automated the measurement of all 40 K_*v*_ channel subtypes (Ranjan et al., 2019), and they used the Data2Dynamics software to fit parameters (Raue et al., 2015). In total, the benchmark contains four references, seven kinetic schemes, and 99 models.

The models were obtained from ModelDB [(McDougal et al., 2017); accession numbers 266925 (AMPA), 50207 (NMDA), and 230137 (Na_*v*_)] and GitHub (K_*v*_).

Some models are numerically unstable with the ame program and were withheld from Table 1. For those models, the propagator matrix was computed with insufficient precision, resulting in a non-monotonic relationship between the number of input partitions and the accuracy of the approximation. The matexp program detected this failure mode and rejected the models.

### 3.4 Accuracy comparison

The integration error of the numerical methods was measured using the analytic solution as the ground truth (see Figure 2). Again, the integration error is the maximum absolute error in any component of the state vector after 1 ms of integration. This was estimated by simulating 10,000 instances of each model in isolation for 1 ms. The state vectors were randomly initialized. The inputs were randomly initialized and held constant thereafter. Identical simulations were run in NEURON using each integration method with a variety of time steps.

**Figure 2:**
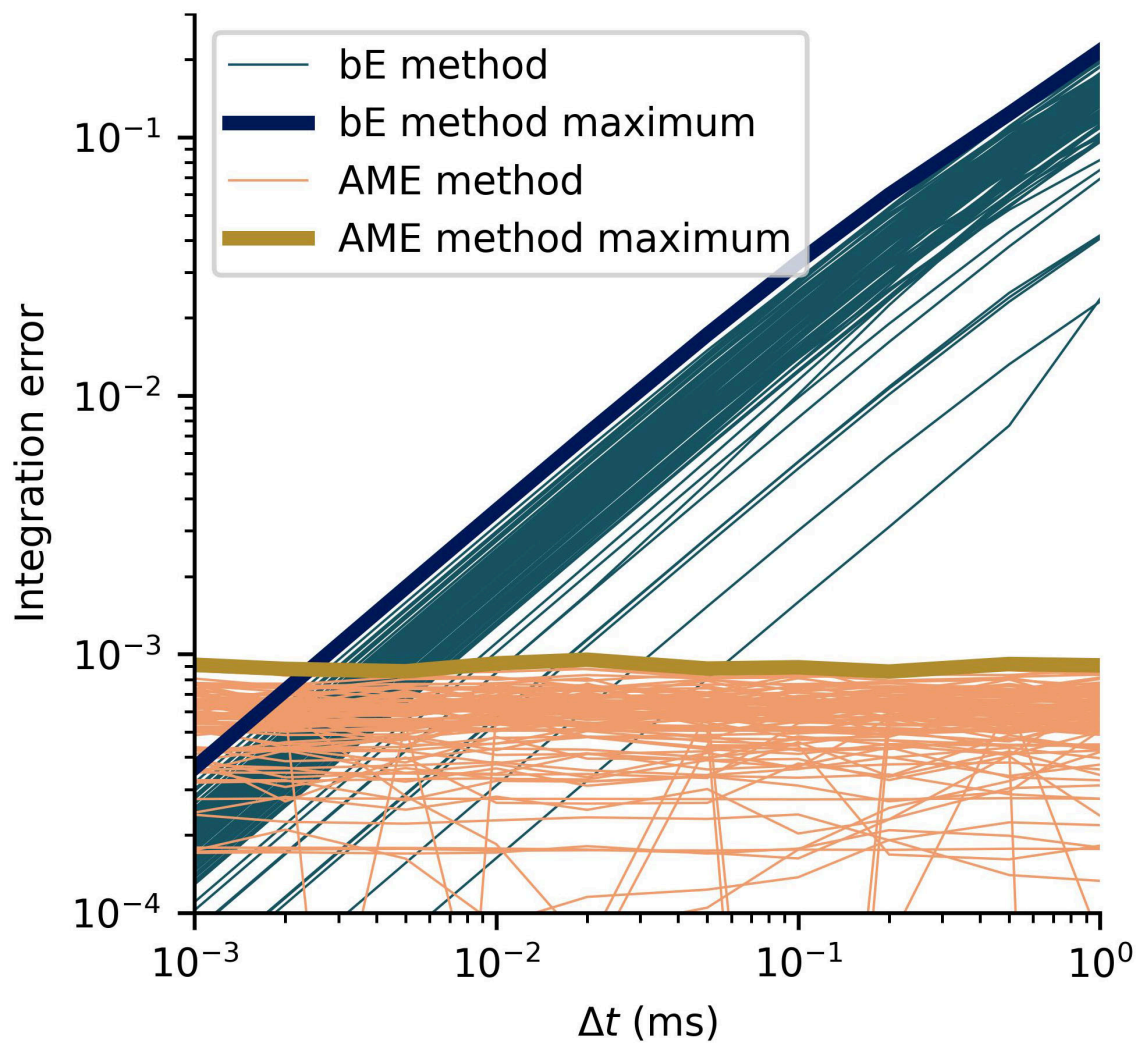
Integration error vs. time step. This compares the bE and AME methods, using the ME method as ground truth. The integration error is the maximum absolute deviation from ground truth after 1 ms of simulation. Each measurement sampled 10,000 random input and state vectors. Thin lines represent models from Table 1. Thick lines indicate the worst observed accuracy for each method over all models. The accuracy of the bE method depends on the time step. The accuracy of the AME method depends on the error target, which was 10^−3^ for this experiment.

The bE method is first order accurate: its error is directly proportional to the time step. In contrast, the AME method controls its accuracy and is not affected by the time step. For this experiment, the AME's error target was 10^−3^, which it achieved for all state and input vectors tested.

### 3.5 Accuracy in context

This section studies how the integration method affects a simulation of an action potential (AP). Because APs broadcast information throughout the brain, AP timing errors quickly cascade. Eight ion channels from Table 1 were combined in a single-compartment neuron to generate APs. The timing of the first AP produced by each integration method under a variety of circumstances was compared (see Figure 3).

**Figure 3:**
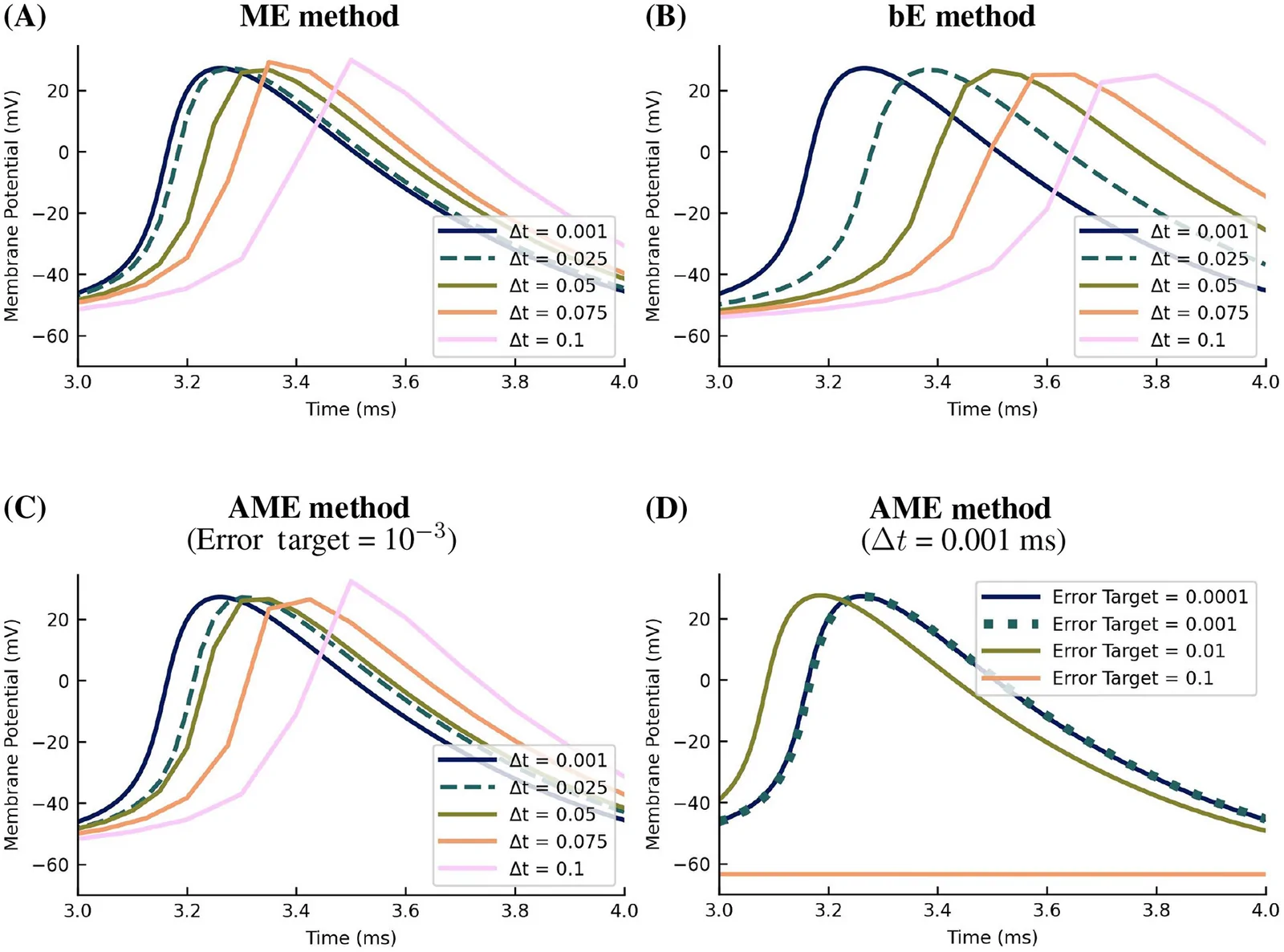
Many simulations of the same AP. This explores how integration method affects a conductance-based neuronal simulation. Using NEURON, eight ion channels from Table 1 were inserted into a single-compartment neuron, which was stimulated by a current clamp to generate an AP. **(A–C)** The simulation was run using each integration method at multiple time steps. **(A)** ME method. **(B)** bE method. **(C)** AME method with an error target of 10^−3^. **(D)** AME method with a time step of 0.001 ms and various error targets. Error targets 0.001 and 0.0001 yielded nearly identical waveforms, indicating convergence. Error target 0.01 generated an AP, despite not converging to the same waveform as higher accuracy simulations. Error target 0.1 was stuck at the resting potential and failed to initiate an AP.

The model neuron had a single compartment with 500 μm^2^ surface area, 1 μF/cm^2^ membrane capacitance, a 0.001 S/cm^2^ leak conductance and the following ion channels inserted: Na_*v*_1.1, Na_*v*_1.4, Na_*v*_1.6, K_*v*_1.1 (6 states), K_*v*_1.2 (6 states), K_*v*_1.6 (6 states), K_*v*_2.1 (11 states), K_*v*_2.2 (6 states). The temperature was 37 °C. The simulation began with a 100 ms initialization period, during which the neuron was silent. After 100 ms a 10 μA current clamp stimulated the neuron for 100 ms, which produced a train of APs.

Four experiments were conducted in NEURON. The first three experiments tested the ME, bE, and AME methods with a linear progression of time steps (0.001 ms, 0.025 ms, 0.05 ms, 0.075 ms, 0.1 ms) and an error target of 10^−3^.

The bE method is first order accurate with respect to the time step and as a result APs are delayed in proportion to the time step. The accuracy of the ME and AME methods is limited by NEURON, which is second order accurate with respect to the time step because it uses the Crank-Nicolson method to solve neuron cable equations. As a result, simulations using the ME and AME methods delay APs in proportion to the square of the time step.

The fourth experiment varied the AME's error target to 10^−4^, 10^−3^, 10^−2^, and 10^−1^ with the time step held constant at 0.001 ms. The AP timing converges as the error target decreases, reaching convergence by an error target of 10^−3^. Error target 10^−2^ yielded APs with small timing errors. The largest error target, 10^−1^, resulted in simulations that outright failed to produce APs.

### 3.6 Speed comparison

The speed of each integration method was measured using NEURON as follows. 1,000 single-compartment neurons were modeled, each containing the following ion channels from Table 1: AMPA, NMDA, Na_*v*_1.1, K_*v*_1.1 (four states) K_*v*_1.1 (six states), K_*v*_1.1 (11 states), and K_*v*_1.1 (13 states). The state vectors were randomly initialized. The input vectors were randomized and held constant. It ran for 1 ms of simulated time at a time step of 0.025 ms and a temperature of 37 °C. The Caliper library (Boehme et al., 2016) measured the time spent advancing each model type.

Ranking the integration methods by run speed always yields the same ordering: in first, second, and third place are the AME, bE, and ME methods, respectively (see Figure 4).

**Figure 4:**
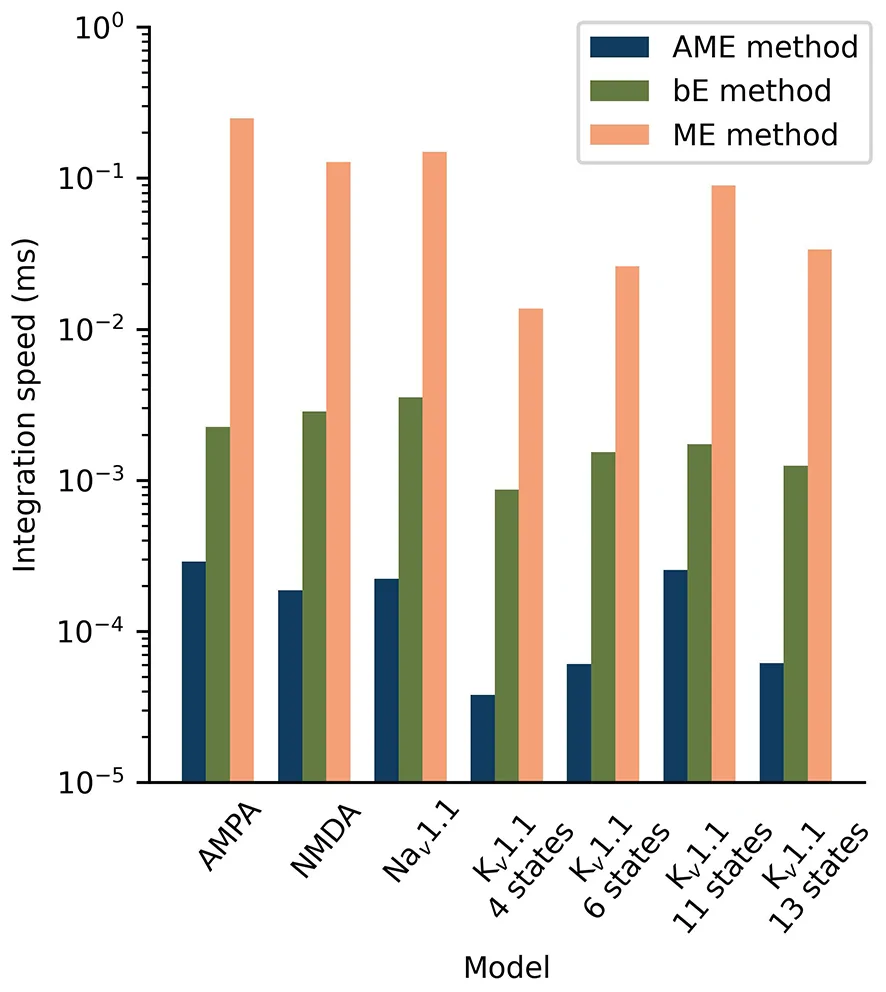
Speed comparison between the bE, ME, and AME integration methods. Lower is better. Using NEURON, seven models from Table 1 were inserted into 1,000 single-compartment neurons and simulated for 1 ms with random initial states, random inputs, a time step of 0.025 ms, and at 37 °C. The AME's error target was 10^−3^. This was performed on an AMD Ryzen 9 9950X3D2.

### 3.7 GPU acceleration

Specialized computers such as GPUs vastly outperform general-purpose computers such as CPUs. The AME method excels on GPUs because its procedure for integration does not contain any conditional branches (e.g., if-statements). The speed of the AME method was measured on a state-of-the-art GPU (see Table 2) using the same methodology as the AME method uses internally to optimize its speed (see Section 2.4). These speeds are attainable only if the GPU runs continuously, which keeps the approximation in cache. Assuming the GPU is dedicated to the task, it can achieve real-time performance for tens to hundreds of thousands of model instances.

**Table 2:** GPU performance evaluation.

Model	Integration speed(ns/step/instance)	Throughput (instances)
AMPA	0.30	82,000
NMDA	1.2	21,000
Na_*v*_1.1	0.11	220,000
K_*v*_1.1 (4 states)	0.059	420,000
K_*v*_1.1 (6 states)	0.096	260,000
K_*v*_1.1 (11 states)	0.21	120,000
K_*v*_1.1 (13 states)	0.29	86,000

Models were solved using the AME method with a time step of 0.025 ms, a temperature of 37 °C, and an error target of 10^−3^. The *Integration speed* column was measured using the same methodology as the AME method uses internally to optimize performance. The *Throughput* column is the maximum number of instances that the GPU can simulate in real time, estimated as the time step divided by the integration speed. This was performed on an NVIDIA B200.

### 3.8 Time complexity

The computational complexity of the AME method is dominated by the piecewise polynomial approximation. Piecewise approximations replace expensive computations with precomputed results, thereby reducing the time complexity to that of accessing memory, which is *not* constant. Computers have hierarchies of caches of varying speeds and sizes. As a result, the time complexity of the AME method is a function of the size of the approximation.

In turn, the size of the approximation is a function of the error target, the time step, and the model. There is a trade-off between speed and accuracy, which is controlled by the error target (see Figure 5). The time step impacts the time complexity in the same way as the error target. The intrinsic difficulty of the model to approximate also impacts the time complexity. The size of the approximation is quadratic with respect to the number of model states, and exponential with respect to the number of input variables. As a result, models with three or more inputs are computationally infeasible.

**Figure 5:**
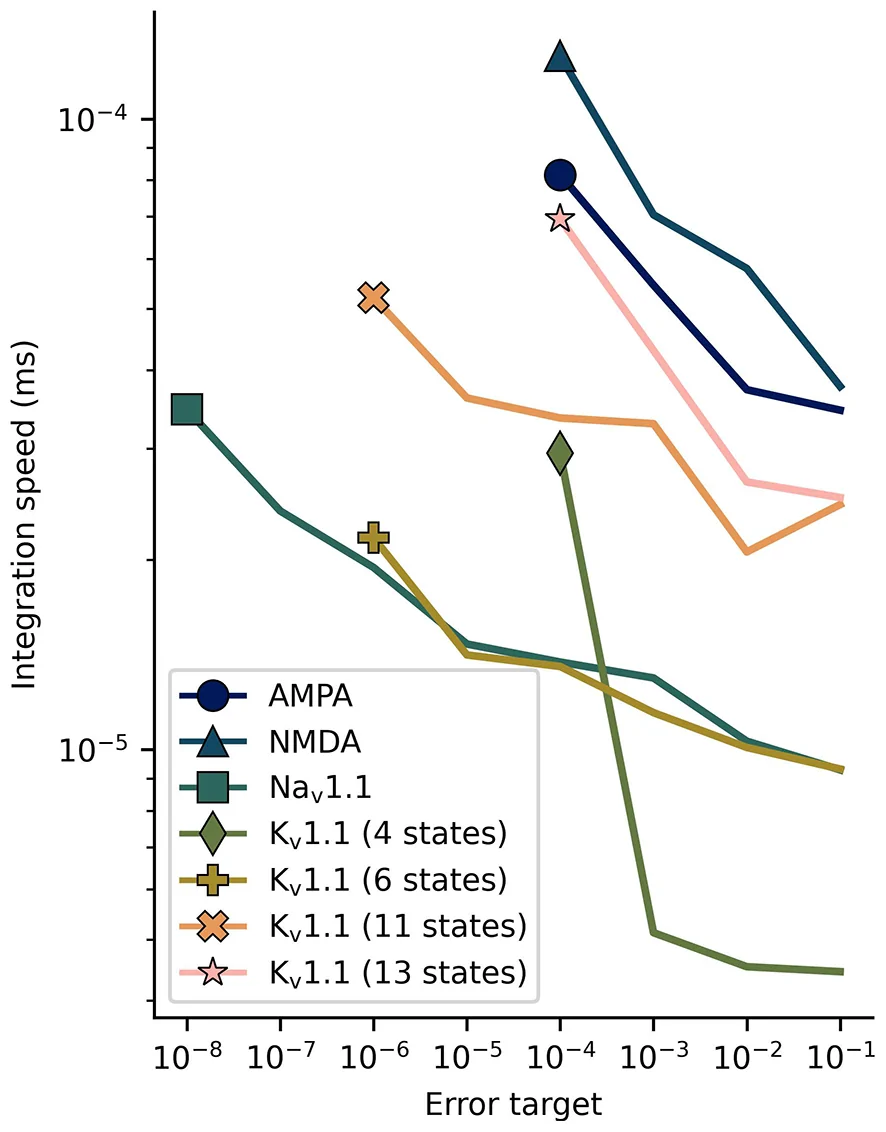
Speed vs. accuracy. The AME method solved seven models from Table 1 with a geometric progression of error targets. For each model, the error target was reduced until the AME method failed due to either excessive runtime or insufficient precision in the matrix exponential calculation. The integration speed was measured using the same methodology as the AME method uses internally. The time step was 0.025 ms, and the temperature was 37 °C.

## 4 Discussion

Markov kinetic models are more expressive than the Hodgkin–Huxley formalism. Markov kinetic models can represent the conformational states of ion channels, whereas Hodgkin–Huxley style models represent only one-dimensional gating variables. However, Markov kinetic models are underutilized in computational neuroscience because they are difficult to construct and simulate. In contrast, the neuroscience literature provides a rich catalog of Hodgkin–Huxley style models, and they have simple analytic solutions. Now, with the advent of fast and accurate simulation methods for Markov kinetic models, it is time for them to take a more prominent role in computational neuroscience.

Future work will need to develop tools for designing Markov kinetic models. Contemporary tools sometimes yield models that are numerically unstable. One cause of numerical instability is large numbers in the transition rate matrix *Q*, which cause large floating-point round-off errors during the matrix exponential computation. For example, transition rate equations which contain exponential functions can be unstable. The solution is to reformulate the models using smaller or bounded transition rates, such as sigmoid (Teed and Silva, 2016) or double sigmoid functions (Balbi et al., 2017).

Future work will also need to compile catalogs of ready-to-use models of ion channels. Ion channels are one of the building blocks of neurons, and so modeling every type of ion channel will enable modeling every type of neuron. The present benchmark is missing many ion channel types and contains many sub-optimal models. In particular, it contains multiple models for each K_*v*_ ion channel subtype, and no attempt has been made to determine which of those models are sufficiently accurate for neuronal simulations. Ideally, a catalog would be either opinionated and pick one model for each type of ion channel, or annotated with notes about the characteristics of each model in order to guide users to the best model for their application.

The AME method is widely applicable despite being limited to two inputs. A large number of commonly modeled ion channels genuinely only have one input, including most voltage-gated ion channels. Ligand-gated ion channels typically bind only one endogenous ligand. However, ligand-gated ion channels bind with many exogenous chemicals, which is incompatible with the AME method. A small number of ion channels accept three or more endogenous inputs, such as NMDA receptors, which accept the membrane's electric potential, the extracellular glutamate concentration, and the extracellular glycine concentration.

The non-zero structure of *Q* does not significantly affect the AME method. The accuracy of the AME method is controlled by the user-defined *error target* parameter and so it is unaffected by the specific state transitions of the kinetic scheme. The speed of the AME method is also largely unaffected by the non-zero structure of *Q* because the AME method utilizes dense matrices throughout. For irreducible Markov chains, *P* is always fully non-zero, and so the AME method's approximation of *P* is likewise dense. Moreover, sparsity generally cannot be exploited over the full range of input values. As a result, the topology of a Markov kinetic scheme does not affect the AME method's applicability.

The time complexity of the AME method is quadratic in the number of model states, which is an improvement over analytic solutions and implicit numerical integration, both of which have cubic time complexity. The AME method is unsuitable for models with a very large number of states, such as molecular dynamics models. If large models can be reduced into smaller, functionally equivalent models, then the AME method can feasibly simulate them.

The AME method decouples accuracy from the time step. The integration error is measured over a 1 ms time period, which was chosen because it is a physiologically relevant time scale for ion channel activity. This allows the error target to be expressed independently of the time step. Furthermore, because the integration error is a worst-case error bound on the state probability, it accumulates linearly. Long term error bounds can be approximated by scaling the error target by the time period of interest.

The AME method is highly accurate and numerically stable. The integration error is essentially random with respect to the input, because correlations between the input and error reset at the boundaries between piecewise regions. Numerical instabilities are limited by the size of the piecewise regions.

A major drawback of the AME method is the substantial computational cost of constructing the approximation. The ame program can take up to an hour to process a single model on a state-of-the-art computer. Once constructed, the approximation can be distributed and reused indefinitely. However, the approximation is optimized for the hardware which performed the ame program's internal performance benchmarks, and so long-term hardware improvements may invalidate the optimizations and necessitate rebuilding and re-optimizing the approximation.

In summary, the AME method offers a unique set of trade-offs. It is both faster and more accurate than the alternative methods evaluated here. However, it is only applicable to Markov kinetic models with one or two inputs and a handful of states. Building and optimizing the approximation is time consuming. It requires a fixed simulation configuration: the temperature, time step, and all PARAMETERS in the NMODL file become CONSTANTS so that they can be baked into the approximation. These are inherent limitations of the method; they cannot be solved, only mitigated. Despite these constraints, this method paves the way for neuron models containing more and more realistic ion channels.

## Data Availability

Publicly available datasets were analyzed in this study. This data can be found here: https://modeldb.science/266925 (AMPA), https://modeldb.science/50207 (NMDA), https://modeldb.science/230137 (Na_*v*_), https://github.com/njohner/Kv-kinetic-models (K_*v*_), and https://doi.org/10.5281/zenodo.20529257 (ame program source-code).
